# New Books

**Published:** 2009-08

**Breakthrough Communities: Sustainability and Justice in the Next American Metropolis**

M. Paloma Pavel, ed.

Cambridge, MA:MIT Press, 2009. 456 pp. ISBN: 978-0-262-01268-3, $54

**Changing Climates in North American Politics: Institutions, Policymaking, and Multilevel Governance**

Henrik Selin, Stacy D. VanDeveer, eds.

Cambridge, MA:MIT Press, 2009. 352 pp. ISBN: 978-0-262-01299-7, $52

**Climate and Society: Climate as Resource, Climate as Risk**

Nico Stehr, Hans von Storch

Hackensack, NJ:World Scientific, 2009. 200 pp. ISBN: 978-981-4280-53-2, $70

**Climate Change and Crops**

S.N.Singe, ed.

New York:Springer, 2009. 384 pp. ISBN: 978-3-540-88245-9, $179

**Science Matters: Humanities as Complex Systems**

Maria Burguete, Lui Lam, eds/

Hackensack, NJ:World Scientific, 2008. 288 pp. ISBN: 978-981-283-593-2, $51

**The Evolution of the Law and Politics of Water**

Joseph W. Dellapenna, Joyeeta Gupta, eds.

New York:Springer, 2009. 413 pp. ISBN: 978-1-4020-9866-6, $169

**The History, Use, Disposition and Environmental Fate of Agent Orange**

Alvin L. Young

New York:Springer, 2009. 339 pp. ISBN: 978-0-387-87485-2, $99

**Water Scarcity, Land Degradation and Desertification in the Mediterranean Region: Environmental and Security Aspects**

J.L. Rubio, U. Safriel, R. Daussa, W.E.H. Blum

New York:Springer, 2009. 156 pp. ISBN: 978-90-481-2525-8, $89.95

**Handbook of Input-Output Economics in Industrial Ecology**

Sangwon Suh

New York:Springer, 2009. 882 pp. ISBN: 978-1-4020-4083-2, $269

**Managers of Global Change: The Influence of International Environmental Bureaucracies**

Frank Biermann, Bernd Siebenhüner, eds.

Cambridge, MA:MIT Press, 2009. 376 pp. ISBN: 978-0-262-01274-4, $56

**Modelling Ocean Climate Variability**

Artem S. Sarkisyan, Jürgen E. Sündermann

New York:Springer, 2009. 374 pp. ISBN: 978-1-4020-9207-7, $179

**Politics of China’s Environmental Protection: Problems and Progress**

Chen Gang

Hackensack, NJ:World Scientific, 2009. 200 pp. ISBN: 978-981-283-869-8, $68

**Counteraction to Chemical and Biological Terrorism in East European Countries**

Christophor Dishovsky, Alexander Pivovarov

New York,:Springer, 2009. 324 pp. ISBN: 978-90-481-2341-4, $89.95

**Crucial Issues in Climate Change and the Kyoto Protocol: Asia and the World**

Kheng-Lian Koh, , Lin Heng Lye, Jolene Lin

Hackensack, NJ:World Scientific, 2009. 550 pp. ISBN: 978-981-4277-52-5, $98

**Global Democracy and Sustainable Jurisprudence: Deliberative Environmental Law**

Walter F. Baber, Robert V. Bartlett

Cambridge, MA:MIT Press, 2009. 248 pp. ISBN: 978-0-262-01302-4, $44

**Governing the Tap: Special District Governance and the New Local Politics of Water**

Megan Mullin

Cambridge, MA:MIT Press, 2009. 280 pp. ISBN: 978-0-262-01313-0, $44

